# SARS-CoV-2 Reinfection Rate and Estimated Effectiveness of the Inactivated Whole Virion Vaccine BBV152 Against Reinfection Among Health Care Workers in New Delhi, India

**DOI:** 10.1001/jamanetworkopen.2021.42210

**Published:** 2022-01-07

**Authors:** Sumit Malhotra, Kalaivani Mani, Rakesh Lodha, Sameer Bakhshi, Vijay Prakash Mathur, Pooja Gupta, Saurabh Kedia, Jeeva Sankar, Parmeshwar Kumar, Arvind Kumar, Vineet Ahuja, Subrata Sinha, Randeep Guleria, and the COVID Reinfection AIIMS Consortium, Aman Dua, Shafi Ahmad, Ramadass Sathiyamoorthy, Ajay Sharma, Tabbu Sakya, Vikas Gaur, Shilpi Chaudhary, Swetambri Sharma, Divya Madan, Anvita Gupta, Shubi Virmani, Arti Gupta, Nidhi Yadav, Surbhi Sachdeva, Shilpi Sharma, Sachin Singh, Abhimanyu Pandey, Mukesh Singh, Divashree Jhurani, Swarnabha Sarkar, Amol Kumar Lokade, Atif Mohammad, Sabitri Pandit, Ritu Dubey, Ajay Kumar Singh, Naveen Gohar, Divyansh Soni, Arunangshu Bhattacharyya, Sabin Rai, Snikitha Tummala, Ishan Gupta, Sakshi Shukla

**Affiliations:** 1 Centre for Community Medicine, All India Institute of Medical Sciences, New Delhi, India; 2 Department of Biostatistics, All India Institute of Medical Sciences, New Delhi, India; 3 Department of Pediatrics, All India Institute of Medical Sciences, New Delhi, India; 4 Department of Medical Oncology, Dr B.R.A Institute–Rotary Cancer Hospital, All India Institute of Medical Sciences, New Delhi, India; 5 Division of Pedodontics and Preventive Dentistry, Centre for Dental Education and Research, All India Institute of Medical Sciences, New Delhi, India.; 6 Department of Pharmacology, All India Institute of Medical Sciences, New Delhi, India; 7 Department of Gastroenterology & Human Nutrition, All India Institute of Medical Sciences, New Delhi, India.; 8 Department of Pediatrics, All India Institute of Medical Sciences, New Delhi, India; 9 Department of Hospital Administration, All India Institute of Medical Sciences, New Delhi, India; 10 Department of Medicine, All India Institute of Medical Sciences, New Delhi, India; 11 Department of Biochemistry, All India Institute of Medical Sciences, New Delhi, India; 12 Department of Pulmonary Critical Care and Sleep Medicine, All India Institute of Medical Sciences, New Delhi, India; 13 Department of Physiology, All India Institute of Medical Sciences, New Delhi, India; 14 Medical Device Monitoring Center, Department of Pharmacology, All India Institute of Medical Sciences, New Delhi, India; 15 Academic Section, All India Institute of Medical Sciences, New Delhi, India

## Abstract

**Importance:**

A surge of COVID-19 occurred from March to June 2021, in New Delhi, India, linked to the B.1.617.2 (Delta) variant of SARS-CoV-2. COVID-19 vaccines were rolled out for health care workers (HCWs) starting in January 2021.

**Objective:**

To assess the incidence density of reinfection among a cohort of HCWs and estimate the effectiveness of the inactivated whole virion vaccine BBV152 against reinfection.

**Design, Setting, and Participants:**

This was a retrospective cohort study among HCWs working at a tertiary care center in New Delhi, India.

**Exposures:**

Vaccination with 0, 1, or 2 doses of BBV152.

**Main Outcomes and Measures:**

The HCWs were categorized as fully vaccinated (with 2 doses and ≥15 days after the second dose), partially vaccinated (with 1 dose or 2 doses with <15 days after the second dose), or unvaccinated. The incidence density of COVID-19 reinfection per 100 person-years was computed, and events from March 3, 2020, to June 18, 2021, were included for analysis. Unadjusted and adjusted hazard ratios (HRs) were estimated using a Cox proportional hazards model. Estimated vaccine effectiveness (1 − adjusted HR) was reported.

**Results:**

Among 15 244 HCWs who participated in the study, 4978 (32.7%) were diagnosed with COVID-19. The mean (SD) age was 36.6 (10.3) years, and 55.0% were male. The reinfection incidence density was 7.26 (95% CI: 6.09-8.66) per 100 person-years (124 HCWs [2.5%], total person follow-up period of 1696 person-years as time at risk). Fully vaccinated HCWs had lower risk of reinfection (HR, 0.14 [95% CI, 0.08-0.23]), symptomatic reinfection (HR, 0.13 [95% CI, 0.07-0.24]), and asymptomatic reinfection (HR, 0.16 [95% CI, 0.05-0.53]) compared with unvaccinated HCWs. Accordingly, among the 3 vaccine categories, reinfection was observed in 60 of 472 (12.7%) of unvaccinated (incidence density, 18.05 per 100 person-years; 95% CI, 14.02-23.25), 39 of 356 (11.0%) of partially vaccinated (incidence density 15.62 per 100 person-years; 95% CI, 11.42-21.38), and 17 of 1089 (1.6%) fully vaccinated (incidence density 2.18 per 100 person-years; 95% CI, 1.35-3.51) HCWs. The estimated effectiveness of BBV152 against reinfection was 86% (95% CI, 77%-92%); symptomatic reinfection, 87% (95% CI, 76%-93%); and asymptomatic reinfection, 84% (95% CI, 47%-95%) among fully vaccinated HCWs. Partial vaccination was not associated with reduced risk of reinfection.

**Conclusions and Relevance:**

These findings suggest that BBV152 was associated with protection against both symptomatic and asymptomatic reinfection in HCWs after a complete vaccination schedule, when the predominant circulating variant was B.1.617.2.

## Key Points

**Question:**

What are the rate of reinfection of SARS-CoV-2 among a cohort of health care workers (HCWs) and the estimated effectiveness of the inactivated whole virion vaccine BBV152 against reinfection?

**Findings:**

In this cohort study of 4978 HCWs who were infected with SARS-CoV-2 from March 3, 2020, to June 18, 2021, the incidence density of reinfection was 7.26 per 100 person-years. A protective association of 86% against reinfection was observed among HCWs who completed the 2-dose schedule of BBV152 and for whom at least 15 days elapsed without reinfection after vaccination.

**Meaning:**

The results of this study suggest that complete vaccination with BBV152 among HCWs in India is crucial, including in persons previously infected with SARS-CoV-2.

## Teaser

This cohort study assesses the SARS-CoV-2 reinfection rate and estimated effectiveness of the inactivated whole virion vaccine BBV152 against reinfection among health care workers in New Delhi, India.

## Introduction

The global COVID-19 pandemic has been ongoing since March 2020.^1^ As of November 23, 2021, more than 257 million COVID-19 cases and 5.15 million fatalities had been reported. India reported the second largest number of cases, 32 285 857, after the US.^2^ India has experienced 2 major COVID-19 waves, with the first major surge reported from August to October 2020, with a maximum reported daily case number of 97 570, and the second surge reported from March to June 2021, with a maximum reported daily case number of 412 262. The second wave recorded a greater magnitude of cases and fatalities, owing to high transmissibility and virulence of the B.1.617.2 (Delta) variant.^3^

Health care workers (HCWs) are at increased risk for SARS-CoV-2 infection as a result of potential occupational exposure.^4,5^ Cases of reinfection among HCWs have also been reported since June 2020.^6,7^ Multiple reasons have been posited for reinfection among HCWs, including persistent occupational exposure; waning natural immunity over time; insufficient seroconversion during a first, milder episode; pandemic fatigue with laxity in personal protection and/or COVID-appropriate behavior; and, possibly, immune escape due to new variants.^8^ Emergency use authorization was awarded to inactivated whole virion vaccine BBV152 (Bharat Biotech Ltd), which is produced in India; this was used in the initial launch of a vaccination program against COVID-19 for HCWs that began on January 16, 2021.^9^ This vaccine requires refrigeration storage at 2 to 8 °C and is available in multidose vials.

Investigating the occurrence of reinfection is imperative with the spread of newer variants (including the Delta variant), and ongoing vaccination programs. Limited information is available on the impact of different vaccines on new infections among previously diagnosed cases, and no previous study estimating the effectiveness of BBV152 on reinfection has been conducted. We report reinfection rates—against a backdrop of the recent second wave and vaccination program from March 3, 2020, to June 18, 2021—among a cohort of HCWs employed at a large tertiary care institution from north India who were previously infected with SARS-CoV-2. We also describe symptoms, symptom severity, and risk factors associated with reinfection episodes.

## Methods

The study was approved by the Institute Ethics Committee of the All India Institute of Medical Sciences, New Delhi, India. Electronic or verbal consent was obtained from the participants because data were collected remotely, and consent procedures adhered to national guidelines for ethics committees reviewing biochemical and health research during the COVID-19 pandemic.^10^ This report follows the Strengthening the Reporting of Observational Studies in Epidemiology (STROBE) reporting guideline.^11^

### Study Setting and Participants

This was a retrospective cohort study conducted among HCWs based at All India Institute of Medical Sciences, a publicly funded teaching and multispecialty tertiary care center in New Delhi, in north India. The cohort comprised HCWs, including both salaried staff and students, for the initiation of a COVID-19 vaccination program within the All India Institute of Medical Sciences beginning on January 16, 2021. Health care workers were offered BBV152 administration within the institute. The vaccination campaign was rolled out via multiple announcements on different platforms. There was a walk-in facility available for all HCWs. Infection events occurring from March 3, 2020, to June 18, 2021, are included in this analysis.

### Study Procedures

We invited all employees (including faculty; scientists and research staff; nursing personnel; and administrative and support staff, including sanitation and security personnel) and students enrolled in the institute in 2020 and 2021 to participate in the study. Dual modalities of data collection were adopted, a web-based electronic form (Google) completed by participants and telephone interview by data collection personnel conducted from May 12 to June 18, 2021. Deaths due to COVID and from non–COVID-related causes were also noted during data collection; telephone interviews of reliable relatives of deceased participants were conducted to enquire about details pertinent to the study, using the verbal autopsy method.^12^ The data collection team was trained extensively in the conduct of telephonic interviews. SARS-CoV-2 positivity and symptom and severity status details, including hospitalization, were collected through self- or family report for all the study participants. All data collected were subjected to stringent quality assurance measures, and all HCWs who reported infection and reinfection were contacted more than once to validate the data.

An epidemiological definition was used for reinfection (ie, any HCW who tested positive for SARS-CoV-2 on 2 separate occasions by either molecular testing (reverse transcription–polymerase chain reaction test, cartridge-based nucleic acid amplification test) or a rapid antigen test), at least 90 days apart.^13^

COVID-19 disease symptoms and severity were graded as asymptomatic or symptomatic. The presence of any of the following was considered symptomatic: fever, rhinorrhea, sore throat, cough, chest pain, wheezing, difficulty breathing, shortness of breath, anosmia, dysgeusia, fatigue, myalgia, headache, abdominal pain, nausea, and diarrhea. Severity of symptomatic infection was categorized as mild, moderate, or severe based on the World Health Organization ordinal scale for clinical improvement.^14^

Health care workers were categorized into 3 groups based on their vaccination status and postvaccination duration, considering the risk for acquiring reinfection in the second wave. Participants were considered fully vaccinated if they received both doses of BBV152 and at least 15 days had elapsed after receipt of the second dose, which was considered maximum protection. Partially vaccinated HCWs received only 1 dose of BBV152 vaccine or received 2 doses but the time between the second dose and the date of data collection or the onset of reinfection was less than 15 days. Unvaccinated HCWs did not receive any doses of BBV152 vaccine.

Health care workers were asked about comorbidities, which included diabetes; hypertension; chronic heart, lung, and kidney diseases; cancer; hypothyroidism; and other self-reported chronic conditions.

### Statistical Analysis

Assuming a reinfection rate among HCWs of 4.5% (based on an Indian archive–based, telephonic survey of individuals infected with SARS-CoV-2),^15^ an absolute precision of 1%, and a nonresponse rate of 15%; the minimum sample required was calculated to be 1943 HCWs infected with SARS-CoV-2. For the estimated vaccine effectiveness study, a sample size of 1657 HCWs was required to achieve 80% predicted vaccine effectiveness with an attack rate of 10% among the unvaccinated.^16^ The desired CI and the absolute precision assumed were 95% and 15%, respectively.

Data management and analysis were done using Stata 16.0 (StataCorp LLC). Quantitative variables were presented as mean and standard deviation. Categorical variables were presented as frequency and percentages. Person-time at risk for reinfection outcome was calculated as follows: the entry time at risk for HCWs was the date of diagnosis of the first episode of infection through a confirmatory test. For those HCWs who were reinfected more than 90 days after their first diagnostic test, the time to the date of diagnosis of the second episode was considered the follow-up period. The rest of the participants were censored on the date of the interview and administration of the study questionnaire during the data collection period from May 12 to June 18, 2021. The incidence density of reinfection (95% CI) was then computed by dividing the number of HCWs who were reinfected with total person-days at risk and presented as 100 person-years.

We estimated the effectiveness of BBV152 according to the vaccine groups. The baseline characteristics were summarized with respect to vaccination risk status using contingency table and χ^2^ test. The probability of reinfection was estimated for vaccination risk status using the Kaplan-Meier method, and the log-rank test was used to compare these probabilities according to vaccination risk status. The Cox proportional hazards model was used to estimate the effectiveness of BBV152 on reinfection, adjusting for significant unbalanced baseline characteristics such as age (categorized as <25, 25-44, and ≥45 years), sex (female and male), type of HCW (faculty, scientist, and/or research staff; nursing staff; junior or senior resident, paramedical and/or support staff; and student or administrative and/or clerical staff). Unadjusted and adjusted hazard ratios (HRs) and 95% CIs are reported for fully vaccinated and partially vaccinated HCWs compared with unvaccinated HCWs. Estimated vaccine effectiveness is given as 1 minus adjusted HR and is reported for overall, symptomatic, and asymptomatic reinfection separately.

In addition to the estimated vaccine effectiveness, we considered the entire cohort of reinfection for determining the risk factors for SARS-CoV-2 reinfection. Both bivariate and multivariate Cox proportional hazards models were used to assess risk factors for reinfection, and the results were reported as HR and 95% CI. A 2-sided *P* ≤ .05 was considered statistically significant.

## Results

**Figure 1.**
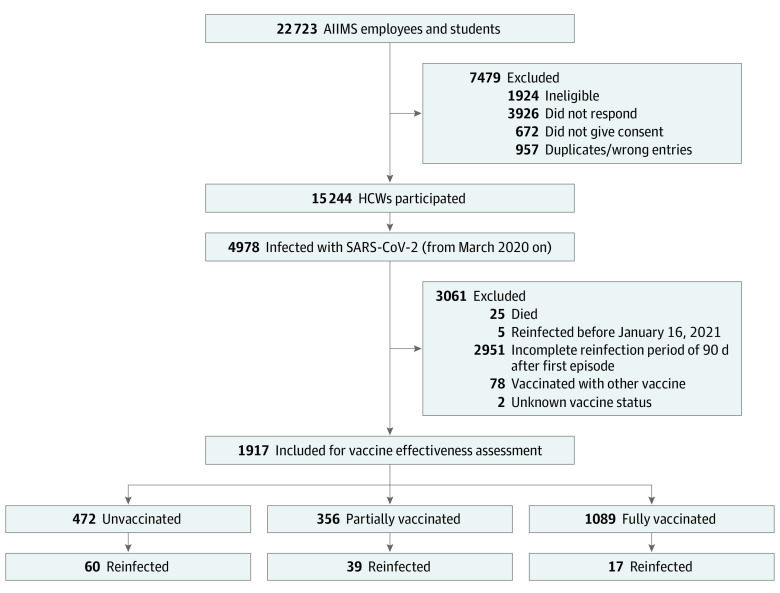
Study Flow Health care workers were divided into 3 vaccine receipt categories: unvaccinated participants did not receive whole virion vaccine BBV152, partially vaccinated participants received only 1 dose of vaccine or had received 2 doses but the interval between second dose and the date of data collection or the onset of reinfection was less than 15 days, and fully vaccinated participants received both doses of vaccine and at least 15 days had elapsed after the receipt of second dose. Of the 124, 116 reinfection events were included in the analysis because 5 were reinfected before vaccine rollout and 3 received a different vaccine. AIIMS indicates All India Institute of Medical Sciences; HCWs, health care workers.

Of the 20 799 eligible HCWs, 15 244 participated in this study (Figure 1). Among these, 4978 participants (32.70%; 95% CI, 31.96%-33.45%; mean [SD] age, 36.6 [10.3] years; and 55.0% male) had at least 1 episode of SARS-CoV-2 infection (eTable 1 in the Supplement). A significantly higher proportion of HCWs aged 45 years or older had moderate to severe disease compared with HCWs aged 44 years or younger (13.2% vs 8.2%, *P *< .001) among HCWs with first-time COVID-19 symptomatic disease. Obesity (body mass index [calculated as weight in kilograms divided by height in meters squared] ≥30) and comorbidity were reported by 9.9% and 20.8% participants, respectively. Twenty-five deaths were recorded; 24 were ascribed to COVID-19 and one to a non–COVID-19 cause; these 25 HCWs were excluded from the estimate of reinfection risk.

Out of the remaining 4953 HCWs, 124 (2.5%) reported a diagnosis of another SARS-CoV-2 episode after a minimum of 90 days between 2 episodes. The median (IQR) interval between 2 infection episodes was 233 (175-321) days (eFigure in the Supplement). The mean (SD) age of those who were reinfected was 35.5 (9.8) years, and 52.9% were male. The reinfection risks according to time period (ie, <180, 180-360, and >360 days) were 13.1% (33 of 252), 5.6% (77 of 1388), and 2.3% (6 of 277), respectively. Compared with less than 180 days, the relative risks of reinfection in the periods 180 to 360 days and greater than 360 days were 0.42 (95% CI, 0.29-0.62) and 0.16 (95% CI, 0.07-0.39), respectively. The total follow-up period given as person-time at risk, contributed by all 4953 HCWs who were previously infected, was 1696 person-years. The incidence density of reinfection in our study cohort was 7.26 (95% CI, 6.09-8.66) per 100 person-years.

### Estimated BBV152 Effectiveness Against Reinfection

For estimating BBV152 effectiveness, 1917 HCWs were included and divided into the 3 vaccine groups: unvaccinated (n = 472), partially vaccinated (n = 356), and fully vaccinated (n = 1089). There were differences in the baseline characteristics among the 3 groups of HCWs (Table 1), and differences in characteristics such as age (*P* < .001), sex (*P* < .001), and type of HCW (*P* < .001) were statistically significant among the 3 groups. Of 1917 HCWs, 1445 (75.3%) were partially vaccinated after they had their first SARS-CoV-2 infection; the median (IQR) duration between the first SAS-CoV-2 infection and vaccination was 183 (131-261) days.

**Table 1.** Demographic and Clinical Characteristics According to Vaccine Risk Category (N = 1917)

Characteristic	No. (%)		*P* value	No. (%)			*P* value
	Total participants	SARS-CoV-2 reinfection		Unvaccinated	Partially	Fully	
No.	1917	116^a^		472	356	1089	
Age, y							
<25	157 (8.2)	15 (12.9)	.08	53 (11.2)	32 (9.0)	72 (6.6)	<.001
25-44	1228 (64.1)	76 (65.5)		335 (71.0)	233 (65.4)	660 (60.6)	
≥45	532 (27.8)	25 (21.6)		84 (17.8)	91 (25.6)	357 (32.8)	
Sex							
Male	1107 (57.7)	62 (53.5)	.33	247 (52.3)	192 (53.9)	668 (61.3)	.001
Female	810 (42.3)	54 (46.5)		225 (47.7)	164 (46.1)	421(38.7)	
Type of HCW							
Student, administrative and/or clerical staff	182 (9.5)	8 (6.9)	.02	49 (10.4)	44 (12.4)	89 (8.2)	<.001
Faculty, scientist, research staff	151 (7.9)	7 (6.0)		34 (7.2)	28 (7.9)	89 (8.2)	
Nursing staff	638 (33.3)	53 (45.7)		193 (40.9)	135 (37.9)	310 (28.5)	
Junior or senior resident	183 (9.6)	14 (12.1)		27 (5.7)	43 (12.1)	113 (10.4)	
Paramedical or support staff	763 (39.8)	34 (29.3)		169 (35.8)	106 (29.8)	488 (44.8)	
BMI							
<18.5	47 (2.4)	2 (1.7)	.60	13 (2.8)	12 (3.4)	22 (2.0)	.23
18.5-24.9	917 (47.8)	61 (52.6)		236 (50.0)	178 (50.0)	503 (46.2)	
25.0-29.9	745 (38.9)	39 (33.6)		168 (35.6)	136 (38.2)	441 (40.5)	
≥30	208 (10.9)	14 (12.1)		55 (11.7)	30 (8.4	123 (11.3)	
Comorbidity^b^	b426 (22.2)	33 (28.5)	.09	110 (23.3)	91 (25.6)	225 (20.7)	.13
First episode COVID-19 symptom status							
Asymptomatic	279 (14.6)	15 (12.9)	.61	82 (17.4)	50 (14.0)	147 (13.5)	.13
Symptomatic	1638 (85.4)	101 (87.1)		390 (82.6)	306 (86.0)	942 (86.5)	

Abbreviations: BMI, body mass index (calculated as weight in kilograms divided by height in meters squared); HCW, health care worker.

^a^ Of 124 reinfection events, 116 were included in the analysis because 5 were reinfected before vaccine rollout and 3 received a different vaccine.

^b^ Defined as diabetes; hypertension; chronic heart, lung, or kidney disease; cancer; hypothyroidism; or other self-reported chronic condition.

**Figure 2.**
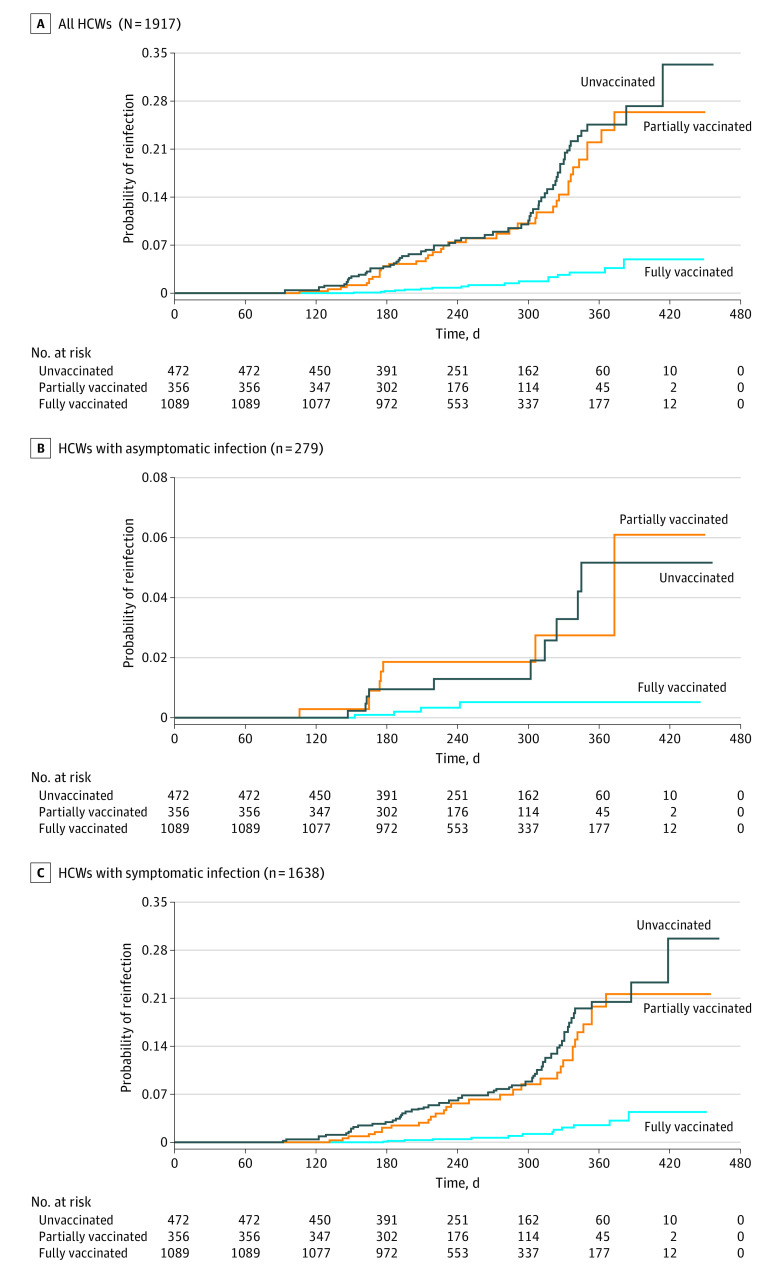
Kaplan-Meier Curve Showing the Probability of Reinfection in the 3 Vaccine Groups, All Participants and According to Symptom Status HCWs indicates health care workers.

Of the 124 who were reinfected, 8 were excluded: 5 were reinfected before vaccine rollout, and 3 received a different vaccine (ChAdOx1-nCOV). Among the 3 vaccine groups, reinfection was reported by 60 (12.7%), 39 (11.0%), and 17 (1.6%) in the unvaccinated, partially vaccinated, and fully vaccinated participants, respectively (Table 2). The corresponding reinfection incidence density was 18.05 (95% CI, 14.02-23.25), 15.62 (95% CI, 11.42-21.38), and 2.18 (95% CI, 1.35-3.51) per 100 person-years, respectively; Figure 2 shows Kaplan-Meier curves of the cumulative probability of reinfection in the 3 groups. The median (IQR) time between partial vaccination and reinfection among these HCWs was 33 (18-62) days. The absolute risk difference between unvaccinated and partially vaccinated was 2.4 per 100 person-years and between unvaccinated and fully vaccinated was 15.8 per 100 person-years.

**Table 2.** Rates of Reinfection With SARS-CoV-2 According to BBV152 Vaccination Status (N = 1917)

BBV152 immunization status	Person-years	SARS-CoV-2 reinfection events		Unadjusted		Adjusted^a^	
		No.	Incidence density per 100 person-years (95% CI)	HR (95% CI)	*P* value	HR (95% CI)	*P* value
**Any reinfection**							
Unvaccinated (n = 472)	333	60	18.05 (14.02-23.25)	1 [Reference]		1 [Reference]	
Vaccinated							
Partially (n = 356)	250	39	15.62 (11.4-21.38)	0.88 (0.59-1.32)	.537	0.88 (0.59-1.32)	.55
Fully (n = 1089)	779	17	2.18 (1.35-3.51)	0.12 (0.07-0.21)	<.001	0.14 (0.08-0.23)	<.001
**Symptomatic reinfection**							
Unvaccinated (n = 472)	333	50	15.04 (11.40-19.84)	1 [Reference]		1 [Reference]	
Vaccinated							
Partially (n = 356)	250	31	12.42 (8.73-17.66)	0.84 (0.53-1.32)	.453	0.84 (0.54-1.33)	.47
Fully (n = 1089)	779	13	1.67 (0.97-2.88)	0.11 (0.06-0.02)	<.001	0.13 (0.07-0.24)	<.001
**Asymptomatic reinfection**							
Unvaccinated (n = 472)	333	10	3.01 (1.62-5.59)	1 [Reference]		1 [Reference]	
Vaccinated							
Partially (n = 356)	250	8	3.20 (1.60-6.41)	1.07 (0.42-2.71)	.885	1.02 (0.40-2.60)	.97
Fully (n = 1089)	779	4	0.51 (0.19-1.37)	0.17 (0.05-0.53)	.002	0.16 (0.05-0.53)	.002

Abbreviation: HR, hazard ratio.

^a^ Adjusted for age, sex, and health worker category.

The adjusted HR for reinfection among fully vaccinated compared with unvaccinated participants was 0.14 (95% CI, 0.08-0.23; *P* < .001); the corresponding estimated BBV152 effectiveness was 86% (95% CI, 77%-92%), whereas it was only 12% and not significant for partially vaccinated compared with unvaccinated (adjusted HR, 0.88; 95% CI, 0.59-1.32; *P* = .55) HCWs. Also, we found significantly greater estimated vaccine effectiveness among fully vaccinated compared with unvaccinated HCWs against symptomatic reinfection (87% [95% CI, 76%-93%]) and asymptomatic reinfection (84% [95% CI, 47%-95%]) (Table 2).

Moderate to severe reinfection occurred in only 6 (0.3%) HCWs (0.3%) in our study cohort. The incidence density in unvaccinated, partially vaccinated, and fully vaccinated participants was 0.23 (95% CI, 0.08-0.72), 0.16 (95% CI, 0.04-0.62), and 0.08 (95% CI, 0.01-0.55), respectively; these differences were not statistically significant. The HRs in the fully vaccinated and partially vaccinated groups were 0.13 (95% CI, 0.01-1.28) and 0.91 (95% CI, 0.15-5.43), respectively, compared with the unvaccinated group.

### Associated Risk Factors

In the multivariable Cox proportional hazard model, age, type of HCW, and comorbidity status were found to be significantly associated with reinfection (Table 3). As compared with HCWs younger than 25 years, the hazards of reinfection were 60% lower for HCWs aged 25 to 44 years (adjusted HR, 0.40; 95% CI, 0.21-0.73; *P* = .003) and 70% lower for HCWs aged 45 years or older (adjusted HR, 0.30; 95% CI, 0.14-0.62; *P* = .001), respectively. For type of HCW, resident physicians had a 3-fold higher hazard (adjusted HR, 2.83; 95% CI; 1.19-6.81; *P* = .02) and nursing staff had a 2-fold higher hazard (adjusted HR, 2.38; 95% CI, 1.05-5.39; *P* = .04) of reinfection, respectively, as compared with students and administrative or clerical staff, who constituted the reference category. Health care workers with comorbidity had a nearly 2 times higher hazard of reinfection compared with HCWs with no comorbidity (adjusted HR, 1.77; 95% CI, 1.15-2.72; *P* = .009).

**Table 3.** Demographic and Clinical Characteristics Associated With COVID-19 Reinfection Among 4953 Previously Infected Health Care Workers Using Cox Proportional Hazards Model

Characteristic	Reinfected, No. (%)		Unadjusted HR (95% CI)	*P* value	Adjusted HR (95% CI)^a^	*P* value
	Yes (n = 124)	No (n = 4829)				
Age, y						
<25	15 (12.1)	444 (9.2)	1 [Reference]		1 [Reference]	
25-44	83 (66.9)	3190 (66.0)	0.62 (0.36-1.07)	.09	0.40 (0.21-0.73)	.003
≥45	26 (21.0)	1195 (24.8)	0.44 (0.23-0.83)	.008	0.30 (0.14-0.62)	.001
Sex						
Female	60 (48.4)	2176 (45.0)	1 [Reference]		1 [Reference]	
Male	64 (51.6)	2653 (55.0)	0.59 (0.41-0.84)	.003	0.97 (0.64-1.47)	.90
Type of HCW						
Student, administrative and/or clerical staff	8 (6.5)	449 (9.3)	1 [Reference]		1 [Reference]	
Faculty, scientist, research staff	7 (5.7)	456 (9.4)	1.09 (0.39-2.99)	.87	1.54 (0.53-4.46)	.43
Nursing staff	56 (45.2)	1690 (35.0)	1.79 (0.85-3.76)	.12	2.38 (1.05-5.39)	.04
Junior or senior resident	18 (14.5)	548 (11.4)	2.29 (0.99-5.27)	.05	2.85 (1.19-6.81)	.02
Paramedical or support staff	35 (28.2)	1686 (34.8)	0.55 (0.25-1.19)	.12	0.72 (0.32-1.63)	.43
BMI						
<18.5	2 (1.6)	124 (2.6)	0.53 (0.13-2.13)	.36	0.55 (0.13-2.27)	.41
18.5-24.9	67 (54.0)	2435 (50.4)	1 [Reference]		1 [Reference]	
25.0-29.9	41 (33.1)	1795 (37.2)	0.80 (0.55-1.19)	.27	0.79 (0.53-1.17)	.23
≥30	14 (11.3)	475 (9.8)	0.96 (0.54-1.70)	.88	1.00 (0.56-1.81)	.99
Comorbidity	36 (29.0)	985 (20.4)	1.50 (1.02-2.22)	.04	1.77 (1.15-2.72)	.009
First episode COVID-19 symptom status						
Asymptomatic	16 (12.9)	525 (10.9)	1 [Reference]		1 [Reference]	
Symptomatic	108 (87.1)	4304 (89.1)	1.23 (0.73-2.08)	.44	1.03 (0.61-1.77)	.90

Abbreviations: BMI, body mass index (calculated as weight in kilograms divided by height in meters squared); HCW, health care worker; HR, hazard ratio.

^a^ Adjusted for age, sex, and health worker category.

### Reinfection Assessment and Symptoms

For most (87%) of the HCWs who were reinfected, the second episode was diagnosed within the institute, predominantly by reverse transcription–polymerase chain reaction test (84%), followed by cartridge-based nucleic acid amplification test (10%). The second episode was reported to be symptomatic by 81% of affected participants, and the median (IQR) symptom duration of the second episode was shorter compared with the first episode (7 [5-12] vs 10 [7-14] days; *P* = .003) (eTable 2 in the Supplement). The median (IQR) duration of hospital stay among hospitalized patients with reinfection (n = 5) was 10 (8-12) days.

## Discussion

This cohort study reports reinfection rates among a large north Indian HCW cohort (n = 4978) with SARS-CoV-2 infection for a 15-month period (March 3, 2020, to June 18, 2021), encompassing 2 waves of the pandemic, with the second surge linked to highly transmissible Delta variant. We identified 124 cases of reinfection (2.5%) with an incidence density of 7.26 (95% CI, 6.09-8.66) per 100 person-years. A previous study from India of the period January 22 to October 7, 2020, reported that out of 1300 individuals, 58 (4.5%) were reinfected.^15^

Low rates of reinfection have been noted in earlier studies among HCWs, other groups, and the general population.^17,18,19,20,21,22^ These studies reported reinfection before 2021 with varying follow-up periods, stages of pandemic, strains of SARS-CoV-2 and its variants, and vaccination use and coverage. In our study, the reinfection risk was highest within the first 180 days and lower thereafter. The vaccination program was rolled out in the study 180 days into the study period, and lower reinfection risk could be ascribed to combined natural infection and vaccination effects.

We observed that BBV152 was associated with a good protective effect (86%) against reinfection in the fully vaccinated group. A similar response was not seen after partial immunization. A study in Kentucky reported that, in May and June of 2021 among persons previously infected with SARS-CoV-2 in 2020, adults who were not vaccinated with an mRNA vaccine had 2.34-fold higher odds of reinfection compared with fully vaccinated adults.^23^

COVID-19 vaccines have been reported to offer protection against variants, including Delta, after completion of the vaccination series, and the effect of partial uptake of vaccines has been found to be suboptimal.^24,25^ In India, BBV152 was found to have overall efficacy of 77.8% (95% CI, 65.2%-86.4%) in a phase 3 trial.^26^ Our estimate of effectiveness against reinfection was similar. Such a high rate of protection might be due to a combination of immune response to both natural infection and vaccine. Neutralization efficacy studies have also reported better performance in BBV152-vaccinated sera compared with sera from recovered but unvaccinated patients.^27,28,29^ Previous laboratory studies found that sera from previously infected persons might have variable and less strong effects against variants of concern, although vaccination could prime the effect and meet the viral infection attack successfully.^30^ The other widely used vaccine in India is the recombinant vaccine ChAdOx1-nCoV (Serum Institute of India Private Limited), and it provides a higher seropositive response to ChAdOx1-nCoV compared with BBV152. This response was associated with comorbidity, sex, vaccine type, and past history of COVID-19 infection.^31^ Early studies suggest a primed immune response in BBV152 recipients to booster doses.^32,33^

In our HCW cohort, older age groups had relatively lower hazards of reinfection than those younger than 25 years; it is possible that older patients had more severe infection in the first episode, which conferred a greater immunogenic response and better protection later.^34^ Nurses and resident physicians are the largest portion of the workforce that has been affected with COVID-19, and the hazards for reinfection were also highest for them in our study, likely owing to repeated exposure opportunities while caring for COVID-19 patients.^35^ Previous studies, largely in the form of case reports and series, have reported different types of comorbidities, reflective of altered immune status, and positive associations with reinfection.^36,37^

No significant differences in symptom status were observed between primary and second infection episodes in our study. Most of those with symptomatic infections had mild disease in the second episode. This is consistent with many reports; however, severe cases also have been reported,^37^ as was in one HCW in our study.

### Strengths and Limitations

This study has strengths, the main ones being the assessment of a protective effect of previous infections and estimation of vaccine effectiveness against reinfection simultaneously among HCWs, with a long follow-up period in a large cohort of HCWs. The study described characteristics of all participants with reinfection and generated evidence after a surge and against a background of the predominant spread of the Delta variant. This is, to our knowledge, 1 of the first studies to report the estimated effectiveness of BBV152 against reinfection in a real-world setting.

This study also has limitations. The second infections in this study are considered possible reinfections because genomic sequencing was not performed for both the episodes, which is required for confirmation of reinfection. Undertaking genomic sequencing for all episodes is logistically impractical owing to resource constraints and has also not been undertaken in earlier reports engaging large numbers of participants.^17^ There is also the possibility of measurement error because the data generated are based on self-reports, although all events were extensively subjected to quality assurance measures. Although a large proportion of testing for confirmation of infection and reinfection occurred within the institute, with testing platforms subjected to internal and external quality standards, we also included laboratory results from outside for pragmatic reasons in a pandemic situation within a low-resource setting. Some of the asymptomatic reinfection cases could have been missed, given that detection was contingent on testing, the rate of which, in the absence of symptoms, would have been lower. Our study population involved working HCWs, and results would be thus applicable to this community. The oldest HCW included in the study was aged 69 years, so assessment of older individuals was not included in the study; older age might have altered the risk of acquiring reinfection and exhibiting vaccine protective responses. Further, in-depth work related to serology and immunologic details are required to correlate the protection offered by previous infection and vaccination. Also, the circumstances regarding reinfection exposures, whether at home or in a hospital setting, were not ascertained. Ours was a single-center, retrospective study, and its generalizability remains to be studied.

## Conclusions

This study found an incidence density of reinfection of 7.3 per 100 person-years among HCWs. These cases occurred after a long follow-up period, of 8 months to 1 year, most notably during the second surge of COVID-19 cases, which was linked to the Delta variant. The inactivated vaccine BBV152 appears to offer a high protective effect of 86% in fully vaccinated HCWs against reinfection. The study generates evidence to vaccinate fully with both the doses, even in HCWs who were previously infected to combat the continuous threat of future surges of SARS-CoV-2 and related variants of concern.

## Notes

Supplement. **eTable 1.** Demographic and Clinical Characteristics of Health Care Workers Who Participated in the Study **eTable 2.** Comparison of First Episode and Second Episode Symptoms and Severity Among COVID-19 Reinfected Health Care Workers (n = 124) **eFigure.** Time Interval Between 2 COVID-19 Episodes
