# Supplementary material for: SARS-CoV-2 Reinfection Rate and Estimated Effectiveness of the Inactivated Whole Virion Vaccine BBV152 Against Reinfection Among Health Care Workers in New Delhi, India
Source: JAMA Netw Open. 2022 Jan 7;5(1):e2142210. doi: 10.1001/jamanetworkopen.2021.42210 (PMC8742193; doi:10.1001/jamanetworkopen.2021.42210)
Supplement: Supplement. — eTable 1. Demographic and Clinical Characteristics of Health Care Workers Who Participated in the Study eTable 2. Comparison of First Episode and Second Episode Symptoms and Severity Among COVID-19 Reinfected Health Care Workers (n = 124) eFigure. Time Interval Between 2 COVID-19 Episodes [file jamanetwopen-e2142210-s001.pdf]

Supplemental Online Content

Malhotra S, Mani K, Lodha R, et al; COVID Reinfection AIIMS Consortium. SARS-CoV-2 reinfection rate and estimated effectiveness of the inactivated whole virion vaccine BBV152 against reinfection among health care workers in New Delhi, India. *JAMA Netw Open*. 2022;5(1):e2142210. doi:10.1001/jamanetworkopen.2021.42210

eTable 1. Demographic and Clinical Characteristics of Health Care Workers Who Participated in the Study

eTable 2. Comparison of First Episode and Second Episode Symptoms and Severity Among COVID-19 Reinfected Health Care Workers (n = 124)

eFigure. Time Interval Between 2 COVID-19 Episodes

This supplemental material has been provided by the authors to give readers additional information about their work.

© 2022 Malhotra S et al. *JAMA Network Open.*

eTable1: Demographic and clinical characteristics of health care workers who participated in the study

	Infected[a] (n=4978)	Not-infected (n=10266)	p-value	Total (n=15244)
**Age in years, mean±SD and n(%)**	36.6 ± 10.3	36.2 ± 11.0		36.3 ± 10.7
< 25	459 (9.2)	1443 (14.1)	< 0.001	
25-44	3276 (65.8)	6147 (59.9)		
≥ 45	1243 (25.0)	2676 (26.1)		
**Sex, n(%)**			<0.001	
Female	2240 (45.0)	3125 (30.4)		5365 (35.2)
Male	2738 (55.0)	7141 (69.6)		9879 (64.8)
**Type of Health Care Worker, n(%)**			< 0.001	
Faculty/Scientist/Research Staff	463 (9.3)	713 (7.0)		1176 (7.7)
Nursing Staff	1746 (35.1)	1573 (15.3)		3319 (21.8)
Junior/Senior Resident	556 (11.4)	626 (6.1)		1192 (7.8)
Paramedical/ Support Staff	1743 (35.0)	6218 (60.6)		7961 (52.2)
Student/ Administrative/Clerical Staff	460 (9.2)	1136 (11.1)		1596 (10.5)
**Body Mass Index (Kg/m²), n(%)**			< 0.001	
< 18·5	127 (2.6)	474 (4.6)		601 (3.9)
18·5-24·9	2511 (50.4)	5777 (56.3)		8288 (54.4)
25·0-29·9	1849 (37.1)	3172 (30.9)		5021 (32.9)
≥ 30	491 (9.9)	843 (8.2)		1334 (8.8)
Comorbidity, n(%)	1035 (20.8)	1494 (14.6)	< 0.001	2529 (16.7)

[a] diagnosed for SARS-CoV-2 by either Reverse Transcription Polymerase Chain Reaction (RT-PCR/ Cartridge Based Nucleic Acid Amplification Test (CBNAAT)/ Rapid Antigen Test (RAT)

© 2022 Malhotra S et al. *JAMA Network Open.*

eTable2. Comparison of first episode and second episode symptoms and severity among COVID-19 reinfected health care workers (n=124)

	First Episode (n=124)	Second Episode (n=124)	p-value
Asymptomatic	16	24	0.13
Symptomatic	108	100	
(n=90)			
Mild	76	84	0.04
Moderate	14	5	
Severe	0	1	
Symptom Duration in days, (n=90)	10 (7-14)	7 (5-12)	0.003
Hospital Duration in days	10 (7-11) (n=36)	10 (8-12) (n=5)	
**Symptoms (n=100)**			
Fever	72	73	0.86
Runny Nose	22	37	0.003
Sore Throat	58	56	0.73
Cough	44	41	0.64
Chest Pain	2	11	0.007
Wheezing	7	8	0.74
Difficulty in Breathing	11	9	0.56
Shortness of Breath	6	7	0.74
Loss of taste	36	27	0.18
Loss of Smell	34	29	0.43
Fatigue	57	66	0.11
Myalgia	51	46	0.34
Headache	38	41	0.63
Abdominal pain	6	7	0.71
Nausea	7	8	0.76
Diarrhea	11	12	0.79

Data are n and median (IQR)

Supplemental Text

First episode was compared with second episode using McNemar's test for paired observations. All the p-values calculated were two-tailed and less than 0.05 was considered statistically significant. The shift in the frequency of asymptomatic infections during the two episodes was not statistically significant during the two episodes (p=0.13). Rhinorrhoea (p=0.003) and chest pain (p=0.007) were more frequent in the second episode. Comparing symptomatic HCWs in both the episodes (n=90), there was significant difference between two episodes, with relatively higher number of mild cases, less of moderate and one severe case reported (p=0.04) during the second episode. The median symptom duration was also lower in the second episode compared to first episode (p=0.003).

© 2022 Malhotra S et al. *JAMA Network Open.*

eFigure. Time interval between two COVID-19 episodes

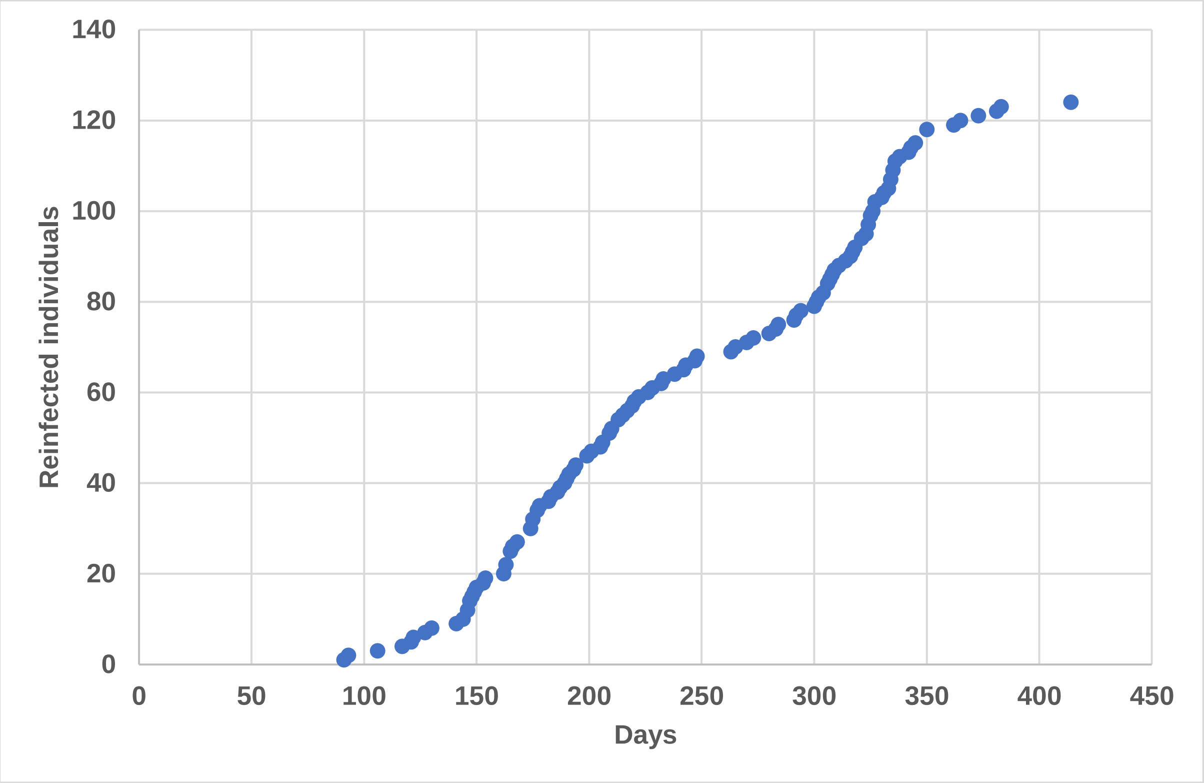

© 2022 Malhotra S et al. *JAMA Network Open.*
